# Integral Valorization of Two-Phase Olive Mill Solid Waste (OMSW) and Related Washing Waters by Anaerobic Co-digestion of OMSW and the Microalga *Raphidocelis subcapitata* Cultivated in These Effluents

**DOI:** 10.1021/acs.jafc.1c08100

**Published:** 2022-03-07

**Authors:** María José Fernández-Rodríguez, David de la Lama-Calvente, Mercedes García-González, José Moreno-Fernández, Antonia Jiménez-Rodríguez, Rafael Borja, Bárbara Rincón-Llorente

**Affiliations:** † Instituto de la Grasa, Consejo Superior de Investigaciones Científicas (CSIC), Campus Universidad Pablo de Olavide, Edificio 46, Carretera de Utrera, km 1, 41013 Sevilla, Spain; ‡ Departamento de Sistemas Físico, Químicos y Naturales, Universidad Pablo de Olavide, Carretera de Utrera, km 1, 41013 Sevilla, Spain; § Instituto de Bioquímica Vegetal y Fotosíntesis (IBVF), Centro de Investigaciones Científicas Isla de la Cartuja, Universidad de Sevilla−Consejo Superior de Investigaciones Científicas (CSIC), Avenida Américo Vespucio 49, 41092 Sevilla, Spain

**Keywords:** anaerobic co-digestion, olive washing water, olive oil washing water, olive mill solid waste, *Raphidocelis subcapitata*, nutrient removal, wastewater treatment, circular economy

## Abstract

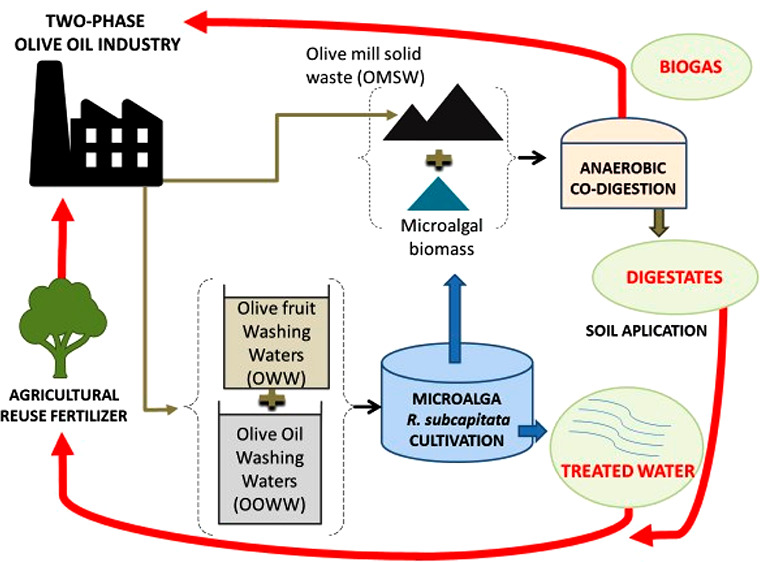

This study evaluates the comprehensive valorization of the byproducts derived from the two-phase olive oil elaboration process [i.e., olive washing water (OWW), olive oil washing water (OOWW), and olive mill solid waste (OMSW)] in a closed-loop process. Initially, the microalga *Raphidocelis subcapitata* was grown using a mixture of OWW and OOWW as the culture medium, allowing phosphate, nitrate, sugars, and soluble chemical oxygen demand removal. In a second step, the microalgal biomass grown in the mixture of washing waters was used as a co-substrate together with OMSW for an anaerobic co-digestion process. The anaerobic co-digestion of the combination of 75% OMSW–25% *R. subcapitata* enhanced the methane yield by 7.0 and 64.5% compared to the anaerobic digestion of the OMSW and *R. subcapitata* individually. This schedule of operation allowed for integration of all of the byproducts generated from the two-phase olive oil elaboration process in a full valorization system and the establishment of a circular economy concept for the olive oil industry.

## 1 Introduction

Olive oil consumption is increasing worldwide as a result of its beneficial health properties. This leads to an increase in olive oil production, not only in countries where olive oil has traditionally been consumed but also in more and more countries, which have begun to produce it.^1^ About 69–80% of the world production comes from the European Union (EU), being the largest producer of olive oil at the global level. The olive tree and its industry are significant activities in the southern member states of the EU, like Spain, Italy, Greece, and Portugal, among others, with important environmental, economic, and social considerations. Spain and Italy are the main producer countries in terms of production, with 63.14 and 17.34% of the EU production, respectively, in the period 2015/2016–2017/2018, followed by Greece with 13.75%.^2^

In the early 1990s, a more sustainable extraction system was implemented. This system, called the “two-phase” system, allowed for the elimination of the wastewaters from the three-phase system, called “alpechines”, which were produced in very high volumes of 1250 L/kg of olives processed and had a high pollutant power, around 200 times more than domestic sewage, as a result of high organic loads.^3^ The two-phase system not only provided the industry with an improvement in the quality of the oil but also decreased the use of water and energy consumption during the process.^4^ Even so, during the more sustainable olive oil production process, three types of byproducts are produced: olive washing water (OWW), which is the effluent from cleaning the olive fruit prior to the production process, olive oil washing water (OOWW), coming from the vertical centrifuge of olive oil cleaning, and finally, a semi-solid waste with high humidity content, generally called olive mill solid waste (OMSW) or alperujo. The OMSW is the main byproduct of the two-phase olive oil process.^4^ The semi-solid byproduct is mainly composed of lignin and cellulose and is characterized by acidic pH, high water content, and elevated organic matter content as a result of the presence of lipids, carbohydrates, phenols, and pectins^5^ as well as a substantial fulvic acid fraction.^6^ The OOWW and OWW are characterized by pH of 5.4 and 6.3, chemical oxygen demand (COD) contents of 15 and 1 g of O_2_/L, total phenol contents of 2400 and 0.4 mg/L, and electrical conductivity of 9.0 and 0.9 mS/cm, respectively.^7^

For each 1000 kg of olives processed by the two-phase olive oil elaboration system, 250 L of a mixture of OWW and OOWW (in a proportion of 1:3) are produced.^8^ Throughout the olive oil production period, between 10 and 15 m^3^ of OOWW and 3–5 m^3^ of OWW are produced on average, each day.^7^

In addition, for each 1000 kg of olives processed, 800 kg of OMSW are produced. This proportion results in 2–4 million tons of OMSW each year.^9^

These byproducts are usually treated as waste. OWW and OOWW are stored in evaporation ponds each season, creating risks of contaminating groundwater with organic loads, generating bad odors, etc.^10,11^ OMSW is also stored in evaporation ponds and burned in co-generation plants.

Despite the great economic impact produced by the olive tree and its industry in Europe and the existence of regulations at the European level concerning the management of waste [Directive 2008/98/EC of the European Parliament and of the Council of 19 November, as amended by Directive (EU) 2018/851 of the European Parliament and of the Council of 30 May 2018], there is no European standard that establishes controls for the handling and treatment of solid and liquid waste from olive oil mills.

In some countries, wastewaters are subjected to advanced wastewater treatments, such as phosphorus adsorption columns, membrane bioreactors, etc., before their discharge from treatment plants. These treatments for nutrient removal are required to meet the quality of surface water, but they are costly and, in some cases, difficult to manage.^12,13^ The production of microalgal biomass coupled to the treatment of wastewaters has been studied by different authors.^14,15^ Microalgal growth in wastewater may offer nutrient removal and be a source of nitrogen-rich biomass.

Anaerobic digestion (AD) is a key tool in support of the circular economy as a result of organic waste being converted into energy.^16^ AD serves to obtain biogas of high calorific value from different organic raw materials. Biogas upgrade has gained interest in recent years because of its use as biofuel for vehicles and for being injected into the natural gas grid.^17^ Furthermore, the liquid digestate obtained after AD can be used as fertilizer.^18^ However, OMSW has a high carbon/nitrogen (C/N) ratio, and although the AD of OMSW is feasible,^9,19,20^ it is possible to obtain higher methane yields by improving the lack of nitrogen in OMSW through the addition of a co-substrate during AD.^21,22^ Xu et al.^23^ reported optimum yields from AD for C/N ratios between 20:1 and 30:1.^23^ The co-digestion of different substrates with complementary composition compensates for the lack of certain elements, which are necessary for the AD process, providing positive synergies between the co-substrates.^24^ Co-digestion not only improves the C/N ratio of the mixture substrates but also improves the enzymatic activity of the bacteria and dilutes the concentration of inhibitory substances in the reactor.^25^

The goal of this work was to valorize the byproducts generated in the two-phase olive oil elaboration process and to assess a closed-loop process for the olive oil industry. To this end, a mixture of OOWW and OWW was used as culture medium for the growth of the microalga *Raphidocelis subcapitata*, and the microalgal biomass produced in this way was used as a co-substrate with OMSW to produce biogas through an AD process. The microalgal growth served to take advantage of the OWW and OOWW, otherwise considered as wastewaters and discharged into evaporation ponds. This scheme valorizes the three byproducts of the two-phase olive oil elaboration process for the first time in an all-inclusive manner. The scheme allows for the production of energy in the form of biogas and for the acquisition of treated water after microalgal growth and a digestate after AD, which can both be used as irrigation and fertilizer with the aim of including the olive oil industry into a circular economy, reusing wastes as byproducts to extend their life and minimizing the industrial impact on the environment.

## 2 Materials and Methods

### 2.1 OMSW, OWW, OOWW, and the Anaerobic Inoculum

The semi-solid byproduct, OMSW, and the two liquid effluents coming from the two-phase olive oil production process, OWW and OOWW, were collected from the Experimental Factory of the “Instituto de la Grasa (CSIC)”, Seville (Spain). The olive stone pieces contained in the OMSW were removed with a 2 mm mesh.

The anaerobic inoculum was obtained from a brewery upflow anaerobic sludge blanket reactor. Table 1 shows the main characteristics of the semi-solid byproduct, OMSW, the two washing waters, OWW and OOWW, and the anaerobic inoculum.

**Table 1:** Characteristics of Olive Oil Washing Water, Olive Washing Water, Olive Mill Solid Waste, and the Microalga *R. subcapitata*^a^

parameter	values of OOWW	values of OWW	values of OMSW	values of *R. subcapitata*	values of inoculum
TS (g L^–1^)	14.8 ± 5.4	4.4 ± 1.1	262.3 ± 1.7^b^	52.6 ± 0.9	33.8 ± 0.4
VS (g L^–1^)	14.6 ± 2.5	2.6 ± 2.4	229.1 ± 2.0^b^	52.1 ± 0.6	27.2 ± 0.6
COD (g of O_2_ L^–1^)	19.4 ± 2.8	2.8 ± 1.5	354.1 ± 4.3^c^	nd	nd
sCOD (g of O_2_ L^–1^)	nd	nd	144.4 ± 4.2	nd	nd
pH	4.7 ± 0.1	10.2 ± 0.3	4.7 ± 0.1	nd	6.9 ± 0.2
C/N ratio	nd	nd	31.6 ± 0.3	8.8 ± 0.2	nd

a OOWW, olive oil washing water; OWW, olive washing water; OMSW, olive mill solid waste; TS, total solids; VS, volatile solids; COD, total chemical oxygen demand; sCOD, soluble chemical oxygen demand; C/N, carbon/nitrogen; and nd, not determined.

b In units of grams per kilogram.

c In units of grams of O_2_ per kilogram.

### 2.2 Microalgal Cultivation

*R. subcapitata* was obtained from the culture collection SAG 61-81.

*R. subcapitata* is a synonym for *Pseudokircheniella subcapitata* (http//:www.algaebase.org). Moreover, the microalgae commonly represented in culture collections as *Selenastrum capricornutum* are not these species but *R. subcapitata*(http//:www.algaebase.org). For this reason, many of the data in this research have been compared to *S. capricornutum*.

Initially, *R. subcapitata* was grown in a 2 L reactor using a modified Arnon medium containing 4 mM K_2_HPO_4_ at pH 7.5 and 25 °C.^26^ Biomass was harvested and, finally, separated by centrifugation for 10 min at 2000*g*. The seed was placed in OOWW, diluted twice with OWW, and supplemented with 15 mM NaNO_3_. The used OOWW/OWW ratio (1:2) and the amount of supplemented NaNO_3_ (15 mM) were selected according to preliminary experimentations. Three reactors were inoculated with the microalga and were held on batch mode for 8 days. The reactors had a capacity of 2 L and were maintained at 25 °C and pH 7.5 and illuminated with white-light lamps (Phillips Master TL5 HO 24 W/840) in a daylight cycle of 12 h of light and 12 h of darkness. The maximum incident irradiance was 1500 μE m^–2^ s^–1^.

Microalgal growth was determined by the chlorophyll measurement as described by Gaur and Kumar.^27^Table 1 shows the main characteristics of the microalgal culture. Washing water samples, OWW and OOWW, were taken every day, and soluble chemical oxygen demand (sCOD) and nitrate, phosphate, and total sugar contents were analyzed to determine nutrient removal from these washing waters.

### 2.3 Biological Methane Potential (BMP) Assays

A mesophilic batch experiment was carried out in 250 mL reactors. The agitation was performed by magnetic bars (22*g*). The BMP tests were operated with different blending ratios of OMSW and microalga at 75% OMSW–25% *R. subcapitata*, 50% OMSW–50% *R. subcapitata*, and 25% OMSW–75% *R. subcapitata* as well as each substrate separately with 100% *R. subcapitata* and 100% OMSW. The inoculum/substrate ratio for each reactor was based on volatile solids (VS) and was 2:1. All of the mixtures were run in triplicate, and three blanks with only inoculum were used as the control. A 1% trace element solution was added as described by Gonzalez-Gil et al.^28^ Reactors were flushed with nitrogen to replace oxygen at the beginning of the experiments. The biogas produced was passed through a 3 N NaOH solution allowing for the measurement of methane production by liquid displacement. When the methane production was lower than 2%, the BMP test was stopped. The methane yield was expressed using the standard temperature and pressure conditions.

### 2.4 Analytical Methods

Nitrate and phosphate were quantified with Hach Lange kits and a Hach Lange DR3900 spectrophotometer. pH was analyzed using a pH-meter model Crison 20 Basic. Total solids (TS) and VS were determined by standard methods 2540B and 2540E, respectively.^29^ COD was determined as described by Raposo et al.^30^ sCOD was evaluated according to standard method 5220D.^29^ Elemental C and N were determined through LECO CHNS-932 (Leco Corporation, St. Joseph, MI, U.S.A.). The total sugar content was determined as described by Dische.^31^

## 3 Results and Discussion

### 3.1 Kinetic Parameters for *R. subcapitata* Growth

#### 3.1.1 Kinetic Parameters for *R. subcapitata* Growth

The Monod equation was used to determine the kinetic parameters of *R. subcapitata* growth and the experimental data from the production of microalgae and nutrient removal in batches.^32,33^

**Figure 1:**
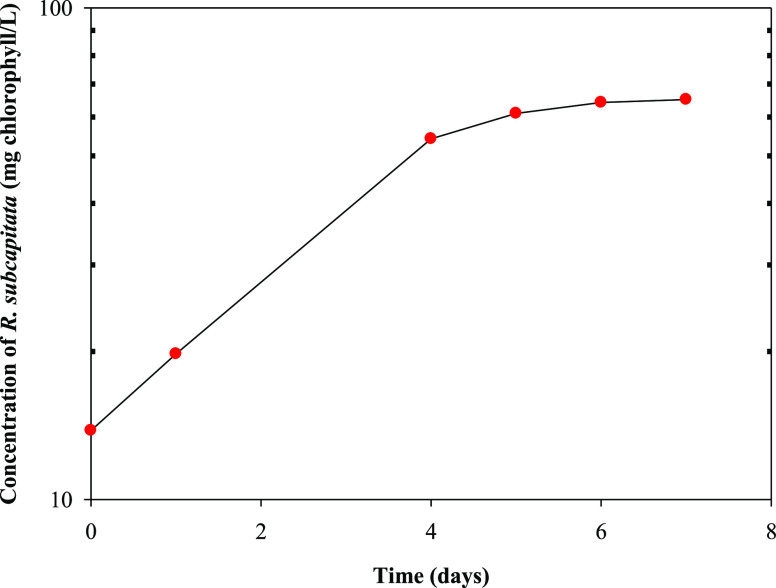
Variation in the concentration of the microalga *R. subcapitata* (mg of chlorophyll L^–1^) with time (day) in the batch culture of microalga in the mixture of washing waters or wastewaters from the olive oil elaboration process.

The starting inoculum of *R. subcapitata* had a concentration of 13.8 mg of chlorophyll L^–1^. The evolution of the concentration of *R. subcapitata* (milligrams of chlorophyll per liter) with cultivation time is shown in Figure 1. As observed, the growth rate drastically decreased after 7 days. The growth decline indicated the end of the experiment. The exponential equation obtained from the integration of the Monod equation could be used to describe *R. subcapitata* biomass growth from 1 to 6 days^34^
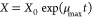
1where μ_max_ is the maximum specific growth rate of the microalga (day^–1^), *X* is the concentration of the microalga in the culture medium (mg of chlorophyll L^–1^), *X*_0_ is the initial concentration of microalga in the culture medium (mg of chlorophyll L^–1^), and *t* is cultivation time (day).

**Figure 2:**
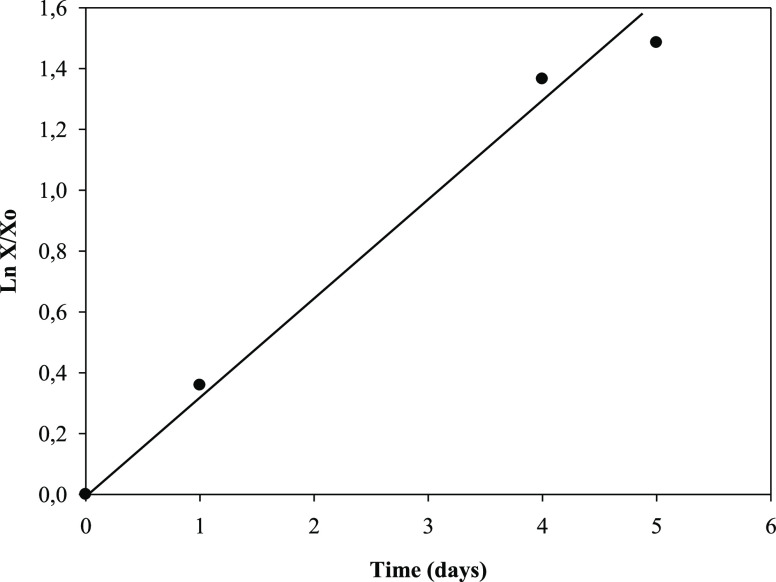
Variation in the ln(*X*/*X*_0_) with cultivation time (day) for μ_max_ calculation in the batch culture of *R. subcapitata* in the mixture of washing waters or wastewaters from the olive oil elaboration process, where μ_max_ is the maximum specific growth rate of the microalga (day^–1^), *X* is the concentration of the microalga in the medium (mg of chlorophyll L^–1^), and *X*_0_ is the initial concentration of the microalga in the culture medium (mg of chlorophyll L^–1^).

Equation 1 can be transformed into eq 2.
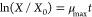
2Therefore, μ_max_ was calculated by adjusting the experimental data from the biomass concentration, ln(*X*/*X*_0_), and cultivation time. Figure 2 shows this linear adjustment to the Monod model, where a satisfactory correlation (*R*^2^ = 0.994; standard error of estimate = 0.098) was obtained. The line slope led to a value of μ_max_ = 0.31 ± 0.02 day^–1^. On the other hand, *g* or the generation time or doubling population time (day)^35^ was estimated by eq 3.
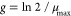
3Considering the value of μ_max_ and according to eq 3, the generation time was 2.23 days.

Similar μ_max_ values were described by Wang et al.^36^ in *S. capricornutum* batch cultures for the simultaneous biogas upgrading and digestate nutrient removal from slurry, regardless of the photoperiod. It was demonstrated that, for photoperiods of 16, 14, and 12 h of light, the maximum specific growth rates were found to be 0.339, 0.341, and 0.326 day^–1^, respectively.^36^

In the same way, similar and lower μ_max_ values than those obtained in the present work were found in batch cultures of *S. capricornutum* when different crude oils were present in the culture medium.^27^

These growth values indicate the ability of *R. subcapitata* to grow in the medium mixture of OWW and OOWW with fairly reasonable growth rates.

#### 3.1.2 Nutrient Removal and Kinetics for Nutrient Removal

**Figure 3:**
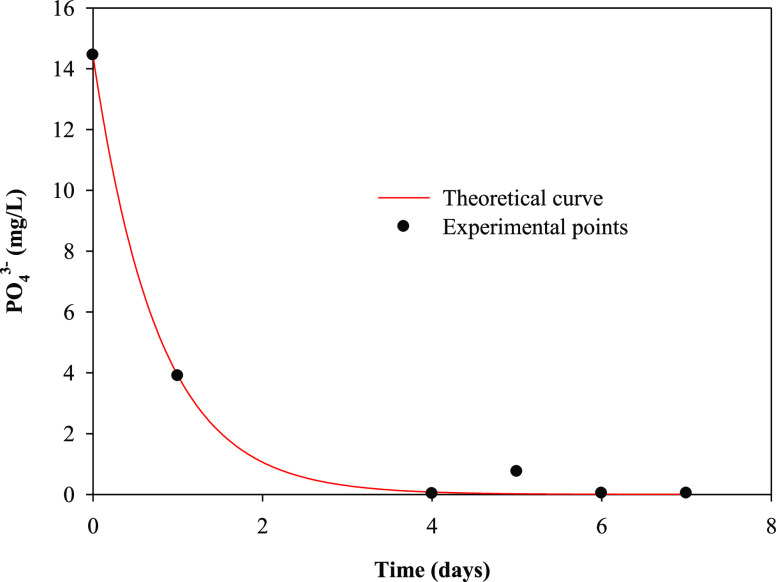
Temporal variation in the phosphate concentration (PO_4_^3–^) and theoretical curve obtained from a pseudo-first-order kinetic model, in the batch culture of *R. subcapitata* in the mixture of washing waters or wastewaters from the olive oil elaboration process.

**Figure 4:**
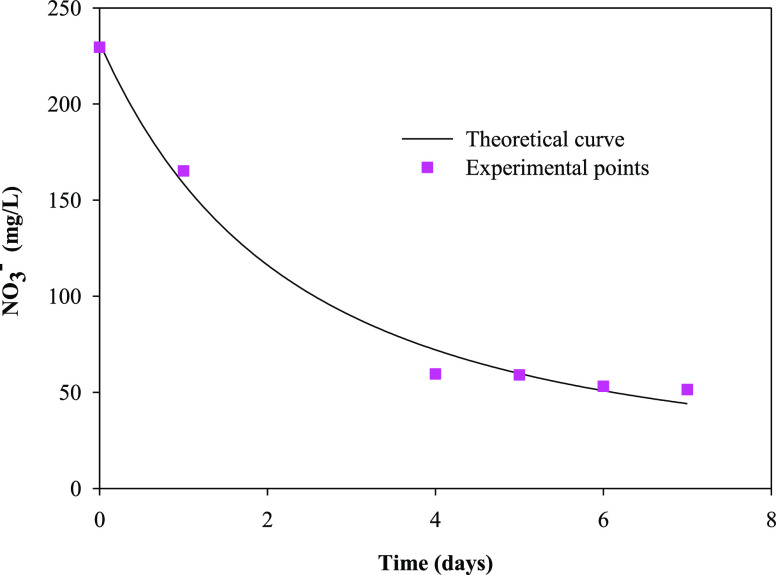
Temporal variation in the nitrate concentration (NO_3_^–^) and theoretical curve obtained from a pseudo-first-order kinetic model in the batch culture of *R. subcapitata* in the mixture of washing waters or wastewaters from the olive oil elaboration process.

**Figure 5:**
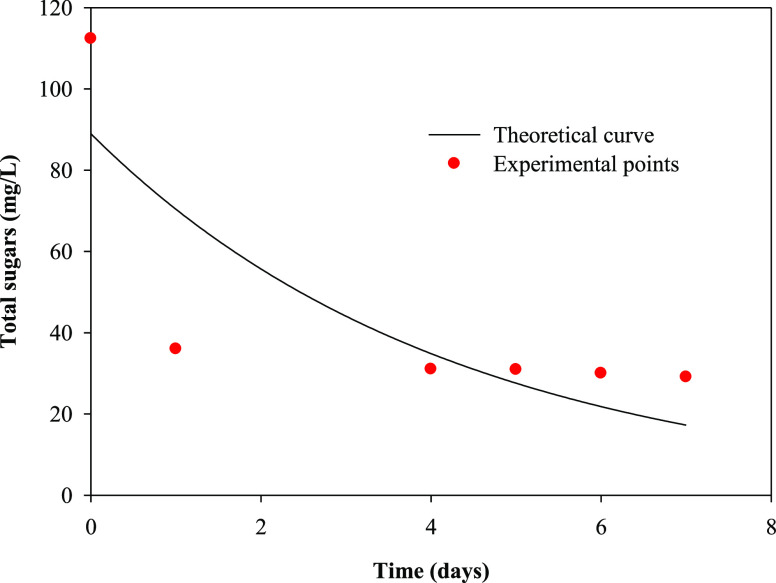
Temporal variation in the total sugar concentration and theoretical curve obtained from a pseudo-first-order kinetic model in the batch culture of *R. subcapitata* in the mixture of washing waters or wastewaters from the olive oil elaboration process.

**Figure 6:**
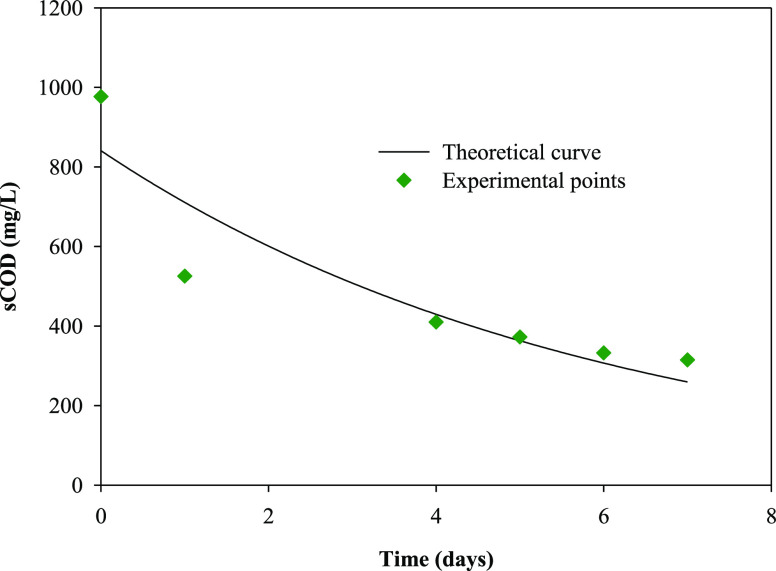
Temporal variation in the sCOD concentration and theoretical curve obtained from a pseudo-first-order kinetic model in the batch culture of *R. subcapitata* in the mixture of washing waters or wastewaters from the olive oil elaboration process.

Figures 3, 4, 5, and 6 show the variation with time of phosphate, nitrate, total sugars, and sCOD concentrations, respectively, in the batch cultures of *R. subcapitata* in the mix of OWW and OOWW used as cultivation medium in the present work. As seen, the maximum percentages of nutrient removal after 7 days of incubation were found to be 99.7, 77.6, 74.1, and 67.8% for phosphate, nitrate, total sugars, and sCOD, respectively.

Microalgae take up nutrient elements (carbon, nitrogen, and phosphorus) for the synthesis of different molecules, such as proteins, nucleic acids, or phospholipids.^37,38^ In systems with pH below 7.5, phosphorus removal mechanisms are given by cellular assimilation because, at this pH, precipitation of phosphorus is not favored.^39^ However, both phosphate and nitrogen removal are linked by their function in microalgal cellular metabolisms. Nitrogen is mostly incorporated into proteins and nucleic acids.^37,39^ Phosphorus uptake, related to storage in the rRNA, is influenced by different factors, such as the phosphate concentration, chemical form of phosphate, algal physiology, nitrogen concentration, light intensity, and temperature.^40^ In this study, phosphate removal (99.7%) was observed to be higher than that observed for nitrate removal (77.6%). However, lower phosphate removals of 35.78 and 40.95% were reported by Wang et al.^36^ for microalga *S. capricornutum* batch cultures. Although it has been reported that, in a control medium, *S. capricornutum* is able to take up phosphate before growing, it was observed that phosphate removal by this microalga could be inhibited by the presence of toxins, such as heavy metals (Pb, Mn, Cr, etc.).^41^

In relation to organic matter removal, Figures 4 and 5 show that total sugars and sCOD removals of 74.1 and 67.8%, respectively, were found in the present work using *R. subcapitata* grown in the mixture of OWW and OOWW as culture medium after 7 days of incubation time. Moreover, similar COD removals (70.3%) were reported by Zhao et al.^33^ when *S. capricornutum* was grown in high-strength synthetic wastewaters and was subjected to high nitrogen loading for 14 days of incubation.

The temporal variation of nutrients in the cultures was described by the pseudo-first-order kinetic model, which can be defined as follows:^12^
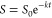
4where *S* is the nutrient concentration at time *t* (day), *S*_0_ is the nutrient concentration at the beginning of the experiment, and *k* is the kinetic constant for nutrient removal. The kinetic constant *k* was determined using the software Sigma Plot (version 11.0). Table 2 shows the pseudo-first-order kinetic parameters for phosphate, nitrate, total sugars, and sCOD removals. The kinetic parameters are essential information for designing biological treatment plants as well as understanding the rate of nutrient and organic matter utilization.^39^Figures 3–6 show the experimental data and theoretical curves obtained for the above-mentioned nutrient and organic matter removals. Table 2 shows the low values for the standard error of estimate and the high values for *R*^2^, indicating the goodness of the fit of the experimental data to the pseudo-first-order kinetic model. The kinetic constant for phosphate removal was 5 times higher than that for nitrate removal and between 5 and 7 times higher than that for total sugars and sCOD removals, respectively.

**Table 2:** Kinetic Parameters Derived from the Application of the Pseudo-First-Order Kinetic Model for Phosphate (PO_4_^3–^), Nitrate (NO_3_^–^), Total Sugars, and sCOD Removals^a^

parameter	*S*_0_ (mg L^–1^)	*k* (day^–1^)	*R*^2^	SEE
PO_4_^3–^	14.4 ± 0.3	1.30 ± 0.09	0.9983	0.367
NO_3_^–^	223 ± 11	0.27 ± 0.02	0.9874	13.439
total sugars	89 ± 18	0.23 ± 0.09	0.9417	22.146
sCOD	840 ± 98	0.17 ± 0.04	0.9584	97.427

a sCOD, soluble chemical oxygen demand; *S*_0_, nutrient concentrations at the beginning; *R*^2^, coefficient of determination; *k*, kinetic constant; and SEE, standard error of estimate.

In relation to phosphate removal, Liu et al.^12^ reported kinetic constant values in the range of 0.93–1.56 day^–1^ using the same kinetic model for the growth of *Chlorella vulgaris* in domestic wastewater. As seen, similar values were found in the present work (1.3 day^–1^), where *R. subcapitata* grew in the mixture of OOWW and OWW. With regard to nitrate removal, Silva et al.^42^ found kinetic constant values in the range of 0.19–0.55 day^–1^ in batch cultures of *C. vulgaris* grown in synthetic media. Similar values are reported in the present work (0.27 day^–1^) with another Chlorophyta as *R. subcapitata*.

There are no reports in the literature regarding kinetic constant values for nutrient removal derived from the aforementioned pseudo-first-order kinetic model in batch cultures of *R. subcapitata* in either synthetic or real wastewaters.

### 3.2 Methane Yields

**Figure 7:**
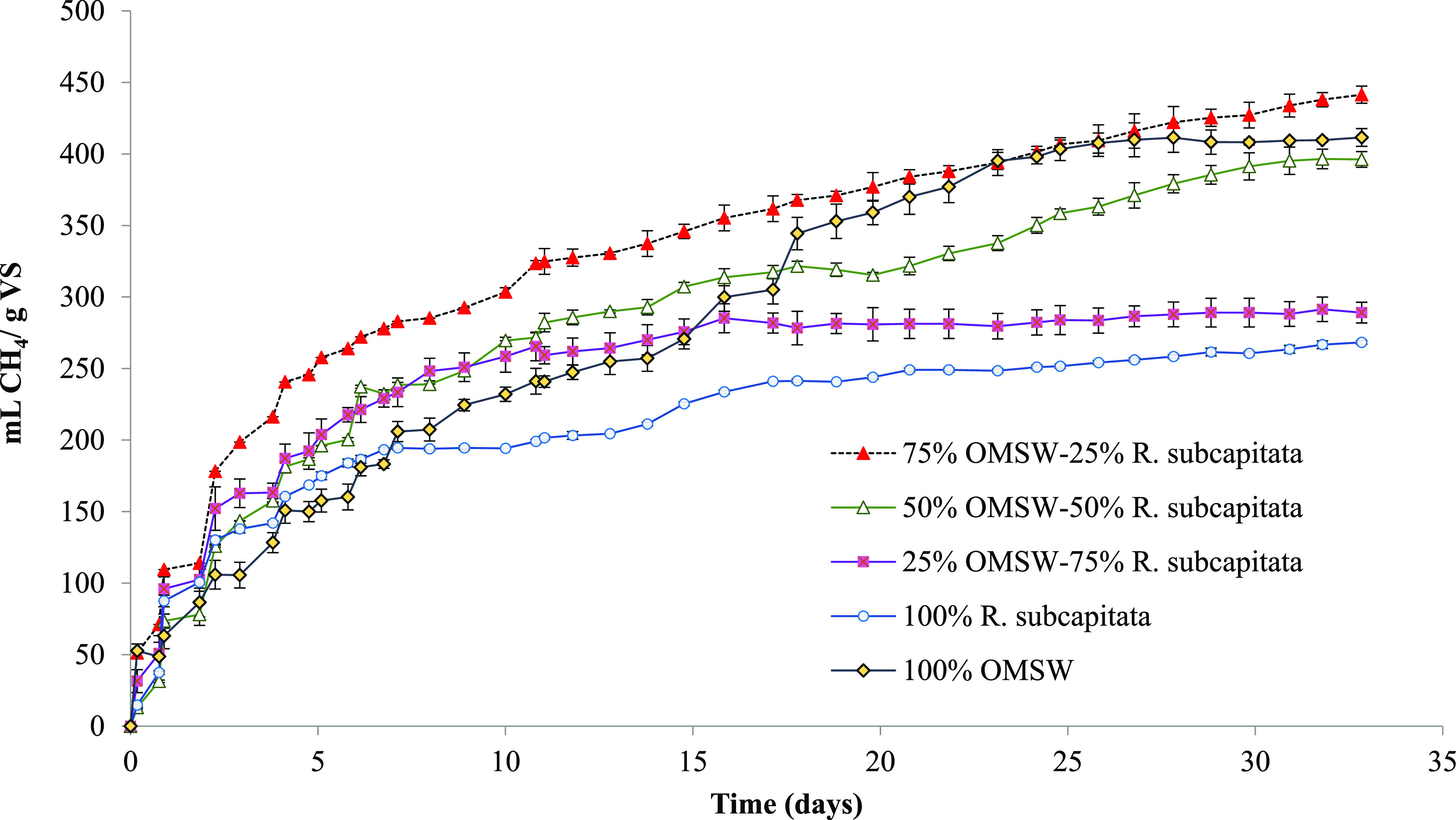
Biochemical methane potential (mL of CH_4_ g^–1^ of VS_added_) of 100% OMSW, 100% *R. subcapitata*, and different co-digestion mixtures.

The variation in specific cumulative methane production with time is illustrated in Figure 7 for the different batch experiment assays.

After 33 days of digestion time, the highest methane yield was 441 mL of CH_4_ g^–1^ of VS_added_ for the co-digestion of the mixture of 75–25% in VS of OMSW and *R. subcapitata*, respectively. The sole substrates reached methane yields of 412 mL of CH_4_ g^–1^ of VS_added_ for the OMSW and 268 mL of CH_4_ g^–1^ of VS_added_ for the AD of *R. subcapitata*. Therefore, the methane yields for the above-mentioned mixture (75% OMSW–25% *R. subcapitata*) were 7.0 and 64.5% higher than that obtained for 100% OMSW and 100% *R. subcapitata*, respectively. Caporgno et al.^43^ found biogas yields of 271 mL of biogas g^–1^ of VS_added_ on the AD of *S. capricornutum*. The methane yield of *R. subcapitata* was also of the same order of magnitude as that found in previous studies on the AD of *Scenedesmus obliquus* (287 mL of CH_4_ g^–1^ of VS_added_).^44^ Analogous results were observed in species characterized by strong cell walls based on carbohydrate compounds.^43^ The methane yields reported by Caporgno et al.^43^ ranged between 330 and 395 mL of biogas g^–1^ of VS_added_ for the co-digestion of 75% sewage sludge–25% *S. capricornutum* and 25% sewage sludge–75% *S. capricornutum*, respectively. A lower methane yield than the value achieved in the present work for the microalga *R. subcapitata* (268 mL of CH_4_ g^–1^ of VS_added_) was recorded by Thorin et al.^45^ in the AD of *S. capricornutum* as a sole substrate (209 mL of CH_4_ g^–1^ of VS_added_). However, when *S. capricornutum* was co-digested with a mixture of waste-activated and primary sludge in a proportion of 75% sludge–25% *S. capricornutum*, the methane yield increased, reaching values of 303 mL of CH_4_ g^–1^ of VS_added_.^45^

The experimental methane yields obtained from the AD of 100% OMSW (412 mL of CH_4_ g^–1^ of VS_added_) and 100% *R. subcapitata* (268 mL of CH_4_ g^–1^ of VS_added_) (Figure 7) were used to obtain the calculated methane yield values (mL of CH_4_ g^–1^ of VS_added_) of the mixtures, according to eq 5
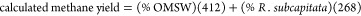
5where % OMSW and % *R. subcapitata* are the percentages of the sole substrates in each co-digestion mixture.

The calculated and experimental methane yields obtained are summarized in Table 3. The experimental methane yield values obtained for the different tests were higher than those calculated by eq 5 across the board, showing synergistic effects (Table 3). The experimental data were 17.3 and 16.4% higher than the calculated data obtained for the co-digestion mixtures of 75% OMSW–25% *R. subcapitata* and 50% OMSW–50% *R. subcapitata*, respectively.

**Table 3:** Calculated Methane Yield Values (eq 5), Experimental Data, and Improvement in Methane Yield with Respect to Its Theoretical Value

OMSW (%)	*R. subcapitata* (%)	calculated (mL of CH_4_ g^–1^ of VS_added_)	experimental (mL of CH_4_ g^–1^ of VS_added_)	improvement (%)
100	0	412	412	0
75	25	376	441	17.3
50	50	340	396	16.4
25	75	304	289	0
0	100	268	268	0

#### 3.2.1 Kinetics of Methane Production

##### 3.2.1.1 First-Order Kinetic Model

A first-order kinetic model was used to assess the anaerobic co-digestion kinetics and evaluate the process performance in the BMP carried out for the three co-digestion mixtures of microalga and OMSW and for OMSW and microalga *R. subcapitata* as sole substrates
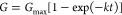
6where *G* is the cumulative specific methane production (mL of CH_4_ g^–1^ of VS_added_), *G*_max_ is the ultimate methane production (mL of CH_4_ g^–1^ of VS_added_), *k* is the specific rate constant (day^–1^), and *t* is the digestion time (day).

BMP tests of biodegradable substrates are usually assessed by this first-order kinetic model.^46^ The main limitation of this model is the proportionality of the methane production and the amount of substrate, which is not limited by the microbiota cell biomass.^47^

**Figure 8:**
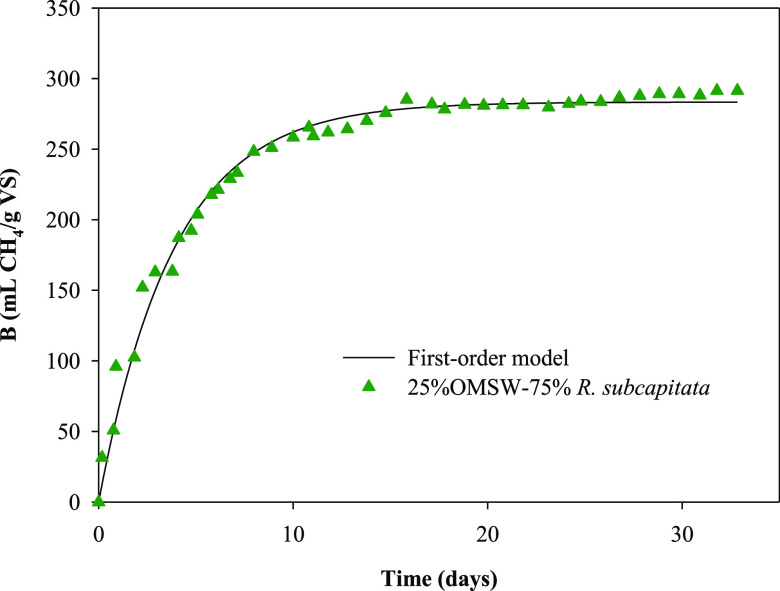
Variation in experimental methane production with time for the mixture of 25% OMSW–75% *R. subcapitata* and the theoretical curve obtained from the first-order kinetic model.

The parameters obtained from the first-order kinetic model (eq 6) are shown in Table 4. The differences between the experimental data and the predicted data are defined as “error”. The errors obtained in this study were lower than 12.4% for all cases. Other evidence of the good fit of the experimentally obtained data to the first-order kinetic model was the low standard deviation values and the high values obtained from the determination coefficients. As an example, Figure 8 shows the experimental methane production data against time for the mixture of 25% OMSW–75% *R. subcapitata* and the theoretical curve of the adjustment to this first-order kinetic model.

**Table 4:** First-Order Kinetic Constant and Ultimate Methane Production (*G*_max_) of the Different Substrates Used: Olive Mill Solid Waste, *R. subcapitata*, and the Different Co-digestion Combinations^a^

substrate	*G*_max_ (mL of CH_4_ g^–1^ of VS_added_)	*k* (day^–1^)	*R*^2^	SEE	error (%)
100% OMSW	461 ± 13	0.07 ± 0.00	0.9882	19.42	12.4
75% OMSW–25% *R. subcapitata*	404 ± 7	0.17 ± 0.01	0.9776	24.50	7.5
50% OMSW–50% *R. subcapitata*	372 ± 6	0.13 ± 0.00	0.9863	18.33	6.0
25% OMSW–75% *R. subcapitata*	283 ± 2	0.26 ± 0.01	0.9925	9.47	2.7
100% *R. subcapitata*	274 ± 4	0.22 ± 0.01	0.9819	16.22	7.8

a OMSW, olive mill solid waste; *G*_max_, experimental values; *k*, kinetic constant; *R*^2^, coefficient of determination; SEE, standard error of estimate; and error, difference between measured and predicted methane yield values.

The values for *G*_max_ are shown in Table 4. These data did not increase with regard to the value found for the OMSW alone when it was co-digested with *R. subcapitata* for the different percentages of mixtures tested. A 271% increase was observed for the kinetic constant, *k*, when the value obtained from the mixture of 75% OMSW–25% *R. subcapitata* was compared to the value obtained for the microalga *R. subcapitata* alone. This increase in *k* was also observed when the value for *k* in the mixture of 75% OMSW–25% *R. subcapitata* was compared to the value obtained for 100% OMSW, although the increase was quite less, at 18%. Syaichurrozi et al.^48^ did not observe any differences in the kinetic constant, *k*, for any of the co-digestion mixtures of *Salvinia molesta* and rice straw. In addition, the values for *k* were considerably smaller (0.01 day^–1^) than the data recorded in the present work (Table 4).

##### 3.2.1.2 Transference Function Model

According to Donoso-Bravo et al.,^49^ the reaction-curve-type model or transference function (TF), used mainly for control purposes, supposes that any process might be considered as an approach that obtains inputs and generates outputs.^49^

The TF model has been used successfully to model the biomethanization of different organic wastes by several authors.^46,49^

The TF model is given by the following expression (eq 7):
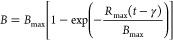
7where *B*_max_ is the ultimate methane production (mL of CH_4_ g^–1^ of VS_added_), *B* is the cumulative specific methane production (mL of CH_4_ g^–1^ of VS_added_), *R*_max_ is the maximum methane production rate (mL of CH_4_ g^–1^ of VS_added_ day^–1^), *t* is the digestion time (day), and γ is lag time (day).

Table 5 shows the kinetic parameters for each experiment and mathematical adjustment. They were determined numerically from the experimental data obtained from nonlinear regression using the software Sigma Plot (version 11).

**Table 5:** Transference Function Model Values for the Different Substrates Used: Olive Mill Solid Waste, *R. subcapitata*, and the Different Co-digestion Mixtures^a^

substrate	*B*_max_ (mL of CH_4_ g^–1^ of VS_added_)	*R*_max_ (mL of CH_4_ (g^–1^ of VS_added_ day^–1^)	γ (day)	*R*^2^	SEE	error (%)
100% OMSW	460 ± 15	34.1 ± 1.9	1.0 × 10^–8^	0.9882	19.67	11.0
75% OMSW–25% *R. subcapitata*	404 ± 7	70.5 ± 4.6	6.6 × 10^–9^	0.9766	24.81	7.5
50% OMSW–-50% *R. subcapitata*	371 ± 6	49.9 ± 2.7	4.3 × 10^–9^	0.9863	18.57	6.3
25% OMSW–75% *R. subcapitata*	283 ± 2	73.3 ± 2.3	1.6 × 10^–11^	0.9925	9.60	2.7
100% *R. subcapitata*	247 ± 4	55.2 ± 4.0	4.3 × 10^–9^	0.9719	16.43	7.8

a OMSW, olive mill solid waste; *B*_max_, ultimate methane production; *R*_max_, maximum methane production rate; γ, calculated lag times; *R*^2^, coefficient of determination; SEE, standard error of estimate; and error, ((*B*_max_ experimental – *B*_max_ model)/*B*_max_ experimental) × 100.

**Figure 9:**
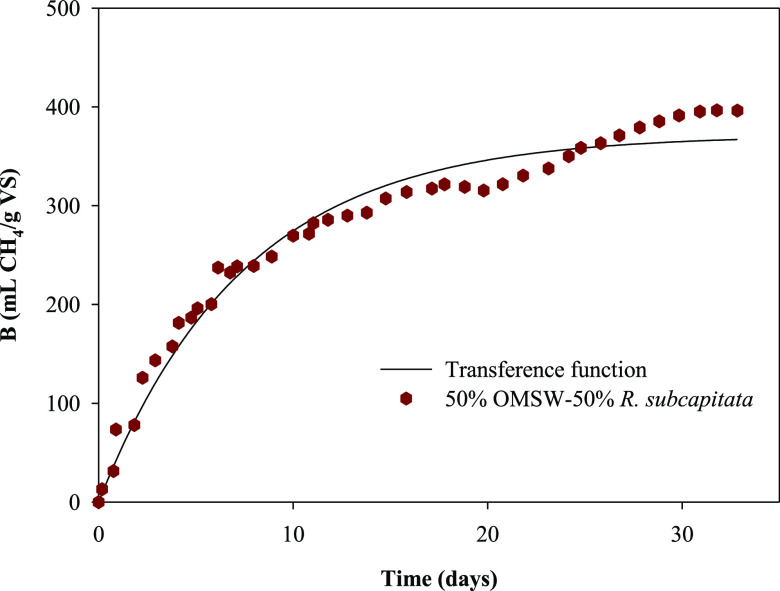
Variation in experimental methane production with time for the mixture of 50% OMSW–50% *R. subcapitata* and the theoretical curve obtained from the transference function model.

Table 5 shows the determination coefficient (*R*^2^), standard error of estimate, and error (%) to evaluate the goodness of fit and accuracy of the results obtained. Error was defined as the difference between the experimental and predicted or theoretical methane yield coefficients in percentage. As seen in Table 5, the high determination coefficient values and the low values for the standard deviations demonstrate the fit of the experimental results to the model. As an example, Figure 9 shows the experimental data for methane production (mL of CH_4_ g^–1^ of VS_added_) versus the digestion time (day) for the mixture of 50% OMSW–50% *R. subcapitata* and the theoretical curve of the adjustment of these points to the TF model.

Among the different co-digestion mixtures assayed, the highest *R*_max_ (73.3 ± 2.3 mL of CH_4_ g^–1^ of VS_added_ day^–1^) was obtained for the co-digestion mixture of 25% OMSW–75% *R. subcapitata*. This value was 3.9 and 46.8% higher than those obtained for 75% OMSW–25% *R. subcapitata* and 50% OMSW–50% *R. subcapitata*, respectively. In addition, it was 114.9 and 32.7% higher than those achieved through the AD of OMSW and *R. subcapitata*, as sole substrates.

On the other hand, a decrease from 73.3 to 55.2 mL of CH_4_ g^–1^ of VS_added_ day^–1^ in *R*_max_ was observed when the content of microalga *R. subcapitata* in the co-digestion mixtures increased from 75 to 100%. Zhang et al.^50^ reported how values for *R*_max_ obtained from the co-digestion of *C. vulgaris* with potato processing waste gradually decreased when the percentages of *C. vulgaris* grew in the mixture from 25 to 75% (on a VS basis).^50^

These results again show how the co-digestion of microalgae with high organic load co-substrates (e.g., food wastes) had a relatively high effect on microalgal biodegradability and conversion rate.

The microalga *R. subcapitata* was able to grow in a mixture of washing waters from the washing of olives and olive oil generated in two-phase olive oil production, showing high potential for removing organic carbon and other nutrients, such as phosphate (99.7%) and nitrate (77.6%), from the mixture. In addition, the anaerobic co-digestion of the mixture of 75% OMSW and 25% *R. subcapitata*, with the microalga *R. subcapitata* being cultivated in the mixture of OWW and OOWW at a 2:1 ratio, showed increases in the methane yield of 7.0 and 64.5% compared to the anaerobic digestion of the sole substrates OMSW and *R. subcapitata*, respectively. The integration of processes, such as microalgal growth, washing water treatment, and AD, allowed for the comprehensive valorization of the byproducts generated in the two-phase olive oil elaboration system, thus closing the loop around this olive oil production system. This approach serves to follow circular economy schedules, which seek the best resource efficiency and environmental management.

## References

[ref1] Bicen P.; Malter A. J. The new institutional economics approach to geographical indication supply chains: A case study from Turkey. In Case Studies in Food Retailing and Distribution; Byrom J., Medway D., Eds.; Woodhead Publishing: Cambridge, U.K., 2019; Chapter 8, pp 105–118, 10.1016/B978-0-08-102037-1.00008-6.

[ref2] European Commission (EC). Study on the Implementation of Conformity Checks in the Olive Oil Sector throughout the European Union; EC: Brussels, Belgium, 2020; 10.2762/274137.

[ref3] Jerman Klen T.; Mozetič Vodopivec B. Ultrasonic Extraction of Phenols from Olive Mill Wastewater: Comparison with Conventional Methods. J. Agric. Food Chem. 2011, 59, 12725–12731. 10.1021/jf202800n. 22053742

[ref4] Alburquerque J. A.; Gonzálvez J.; García D.; Cegarra J. Agrochemical characterization of “alperujo”, a solid by-product of the two-phase centrifugation method for olive oil extraction. Bioresour. Technol. 2004, 91, 195–200. 10.1016/S0960-8524(03)00177-9. 14592750

[ref5] Maragkaki A. E.; Vasileiadis I.; Fountoulakis M.; Kyriakou A.; Lasaridi K.; Manios T. Improving biogas production from anaerobic co-digestion of sewage sludge with a thermal dried mixture of food waste, cheese whey and olive mill wastewater. Waste Manage. 2018, 71, 644–651. 10.1016/j.wasman.2017.08.016. 28807555

[ref6] Rodríguez-Lucena P.; Hernández D.; Hernández-Apaolaza L.; Lucena J. J. Revalorization of a two-Phase olive mill waste extract into a micronutrient fertilizer. J. Agric. Food Chem. 2010, 58, 1085–1092. 10.1021/jf903185z. 20028019

[ref7] Ochando-Pulido J. M.; Hodaifa G.; Victor-Ortega M. D.; Rodriguez-Vives S.; Martinez-Ferez A. Reuse of olive mill effluents from two-phase extraction process by integrated advanced oxidation and reverse osmosis treatment. J. Hazard. Mater. 2013, 263, 158–167. 10.1016/j.jhazmat.2013.07.015. 23910394

[ref8] Alba J.; Ruiz M. A.; Hidalgo F.; Martínez F.; Moyano M. J.; Borja R.; Graciani E.; Ruiz M. V. Elaboración del aceite de oliva virgin. In El Cultivo del Olivo, 4th ed.; Barranco D., Fernández-Escobar R., Rallo L., Eds.; Mundi Prensa Libros, S.A.: Madrid, Spain, 2001.

[ref9] Rincón B.; Bujalance L.; Fermoso F. G.; Martín A.; Borja R. Biochemical methane potential of two-phase olive mill solid waste: Influence of thermal pretreatment on the process kinetics. Bioresour. Technol. 2013, 140, 249–255. 10.1016/j.biortech.2013.04.090. 23707912

[ref10] Karaouzas I. Agro-Industrial Wastewater Pollution in Greek River Ecosystems. In The Rivers of Greece. The Handbook of Environmental Chemistry; Skoulikidis N., Dimitriou E., Karaouzas I., Eds.; Springer: Berlin, Germany, 2016; Vol. 59, pp 169–204, 10.1007/698_2016_453.

[ref11] Sáez J. A.; Pérez-Murcia M. D.; Vico A.; Martínez-Gallardo M. R.; Andreu-Rodríguez F. J.; López M. J.; Bustamante M. A.; Sanchez-Hernandez J. C.; Moreno J.; Moral R. Olive mill wastewater-evaporation ponds long term stored: Integrated assessment of in situ bioremediation strategies based on composting and vermicomposting. J. Hazard. Mater. 2021, 402, 123481 10.1016/j.jhazmat.2020.123481. 32736177

[ref12] Liu X.; Ying K.; Chen G.; Zhou C.; Zhang W.; Zhang X.; Cai Z.; Holmes T.; Tao Y. Growth of *Chlorella vulgaris* and nutrient removal in the wastewater in response to intermittent carbon dioxide. Chemosphere 2017, 186, 977–985. 10.1016/j.chemosphere.2017.07.160. 28835006

[ref13] Oller I.; Malato S.; Sánchez-Pérez J. A. Combination of advanced oxidation processes and biological treatments for wastewater decontamination—A review. Sci. Total Environ. 2011, 409, 4141–4166. 10.1016/j.scitotenv.2010.08.061. 20956012

[ref14] Rasoul-Amini S.; Montazeri-Najafabady N.; Shaker S.; Safari A.; Kazemi A.; Mousavi P.; Mobasher M. A.; Ghasemi Y. Removal of nitrogen and phosphorus from wastewater using microalgae free cells in bath culture system. Biocatal. Agric. Biotechnol. 2014, 3, 126–131. 10.1016/j.bcab.2013.09.003.

[ref15] Acién Fernández F. G.; Gómez-Serrano C.; Fernández-Sevilla J. M. Recovery of nutrients from wastewaters using microalgae. Front. Sustain. Food Syst. 2018, 2, 59 10.3389/fsufs.2018.00059.

[ref16] Abad V.; Avila R.; Vicent T.; Font X. Promoting circular economy in the surroundings of an organic fraction of municipal solid waste anaerobic digestion treatment plant: Biogas production impact and economic factors. Bioresour. Technol. 2019, 283, 10–17. 10.1016/j.biortech.2019.03.064. 30897388

[ref17] Börjesson P.; Mattiasson B. Biogas as a resource-efficient vehicle fuel. Trends Biotechnol. 2008, 26, 7–13. 10.1016/j.tibtech.2007.09.007. 18036686

[ref18] Iocoli G. A.; Zabaloy M. C.; Pasdevicelli G.; Gómez M. A. Use of biogas digestates obtained by anaerobic digestion and co-digestion as fertilizers: Characterization, soil biological activity and growth dynamic of *Lactuca sativa* L. Sci. Total Environ. 2019, 647, 11–19. 10.1016/j.scitotenv.2018.07.444. 30077158

[ref19] Borja R.; Rincón B.; Raposo F.; Alba J.; Martín A. A study of anaerobic digestibility of two-phases olive mill solid waste (OMSW) at mesophilic temperature. Process Biochem. 2002, 38, 733–742. 10.1016/S0032-9592(02)00202-9.

[ref20] Borja R.; Martín A.; Rincón B.; Raposo F. Kinetics for substrate utilization and methane production during the mesophilic anaerobic digestion of two phases olive pomace (TPOP). J. Agric. Food Chem. 2003, 51, 3390–3395. 10.1021/jf021059n. 12744672

[ref21] Fernández-Rodríguez M. J.; Rincón B.; Fermoso F. G.; Jiménez A. M.; Borja R. Assessment of two-phase olive mill solid waste and microalgae co-digestion to improve methane production and process kinetics. Bioresour. Technol. 2014, 157, 263–269. 10.1016/j.biortech.2014.01.096. 24561632

[ref22] Fernández-Rodríguez M. J.; de la Lama-Calvente D.; Jiménez-Rodríguez A.; Borja R.; Rincón-Llorente B. Influence of the cell wall of *chlamydomonas reinhardtii* on anaerobic digestion yield and on its anaerobic co-digestion with a carbon-rich substrate. Process Saf. Environ. Protect. 2019, 128, 167–175. 10.1016/j.psep.2019.05.041.

[ref23] Xu J.; Wang X.; Sun S.; Zhao Y.; Hu C. Effects of influent C/N ratios and treatment technologies on integral biogas upgrading and pollutants removal from synthetic domestic sewage. Sci. Rep. 2017, 7, 10897 10.1038/s41598-017-11207-y. 28883448 PMC5589932

[ref24] Ma X.; Yu M.; Yang M.; Gao M.; Wu C.; Wang Q. Synergistic effect from anaerobic co-digestion of food waste and *Sophora flavescens* residues at different co-substrate ratios. Environ. Sci. Pollut. Res. Int. 2019, 26, 37114–37124. 10.1007/s11356-019-06399-x. 31745798

[ref25] Barua V. B.; Rathore V.; Kalamdhad A. S. Anaerobic co-digestion of water hyacinth and banana peels with and without thermal pretreatment. Renew. Energy 2019, 134, 103–112. 10.1016/j.renene.2018.11.018.

[ref26] Arnon D. I.; McSwain B. D.; Tsujimoto H. Y.; Wada K. Photochemical activity and components of membrane preparations from blue-green algae. I. Coexistence of two photosystems in relation to chlorophyll a and removal of phycocyanin. Biochim. Biophys. Acta-Bioenerg. 1974, 357, 231–245. 10.1016/0005-2728(74)90063-2. 4153919

[ref27] Gaur J. P.; Kumar H. D. Growth response of four micro-algae to three crude oils and a furnace oil.. Environ. Pollut. Series A, Ecological and Biological 1981, 25, 77–85. 10.1016/0143-1471(81)90116-1.

[ref28] Gonzalez-Gil G.; Seghezzo L.; Lettinga G.; Kleerebezem R. Kinetics and mass-transfer phenomena in anaerobic granular sludge. Biotechnol. Bioeng. 2001, 73, 125–134. 10.1002/bit.1044. 11255160

[ref29] American Public Health Association (APHA), American Water Works Association (AWWA), and Water Environment Federation (WEF). Standard Methods for the Examination of Water and Wastewater; APHA, AWWA, and WEF: Washington, D.C., 2012.

[ref30] Raposo F.; de la Rubia M. A.; Borja R.; Alaiz M. Assessment of a modified and optimized method for determining chemical oxygen demand of solid substrates and solutions with high suspended solid content. Talanta 2008, 76, 448–453. 10.1016/j.talanta.2008.03.030. 18585304

[ref31] Dische Z. Color reactions of carbohydrates. In Methods in Carbohydrates Chemistry; Whistler R. L., Wolfram M. L., Eds.; Academic Press: New York, 1962; pp 477–512.

[ref32] Molazadeh M.; Danesh S.; Ahmadzadeh H.; Pourianfar H. R. Influence of CO_2_ concentration and N:P ratio on Chlorella vulgaris-assisted nutrient bioremediation, CO_2_ biofixation and biomass production in a lagoon treatment plant. J. Taiwan Inst. Chem. Eng. 2019, 96, 114–120. 10.1016/j.jtice.2019.01.005.

[ref33] Zhao Y.; Ge Z.; Lui H.; Sun S. Ability of different microalgae species in synthetic high-strength wastewater treatment and potential lipid production. J. Chem. Technol. Biotechnol. 2016, 91, 2888–2895. 10.1002/jctb.4905.

[ref34] Rodríguez R.; Espada J. J.; Moreno J.; Vicente G.; Bautista L. F.; Morales V.; Sánchez-Bayo A.; Dufour J. Environmental analysis of *Spirulina* cultivation and biogas production using experimental and simulation approach. Renewable Energy 2018, 129, 724–732. 10.1016/j.renene.2017.05.076.

[ref35] Machado M. D.; Soares E. V. Impact of erythromycin on a non-target organism: Cellular effects on the freshwater microalga *Pseudokirchneriella subcapitata*. Aquat. Toxicol. 2019, 208, 179–186. 10.1016/j.aquatox.2019.01.014. 30682620

[ref36] Wang Z.; Zhao Y.; Ge Z.; Zhang H.; Sun S. Selection of microalgae for simultaneous biogas upgrading and biogas slurry nutrient reduction under various photoperiods. J. Chem. Technol. Biotechnol. 2016, 91, 1982–1989. 10.1002/jctb.4788.

[ref37] Kumar A.; Ergas S.; Yuan X.; Sahu A.; Zhang Q.; Dewulf J.; Malcata F. X.; van Langenhove H. Enhanced CO_2_ fixation and biofuel production via microalgae: Recent developments and future directions. Trends Biotechnol. 2010, 28, 371–380. 10.1016/j.tibtech.2010.04.004. 20541270

[ref38] Yan C.; Zhao Y.; Zheng Z.; Luo X. Effects of various LED light wavelengths and light intensity supply strategies on synthetic high-strength wastewater purification by *Chlorella vulgaris*. Biodegradation 2013, 24, 721–32. 10.1007/s10532-013-9620-y. 23371421

[ref39] Katam K.; Bhattacharyya D. Comparative study on treatment of kitchen wastewater using a mixed microalgal culture and an aerobic bacterial culture: Kinetic evaluation and FAME analysis. Environ. Sci. Pollut. Res. 2018, 25, 20732–20742. 10.1007/s11356-018-2209-6. 29754302

[ref40] Gupta P. L.; Choi H. J.; Lee S. M. Enhanced nutrient removal from municipal wastewater assisted by mixotrophic microalgal cultivation using glycerol. Environ. Sci. Pollut Res. 2016, 23, 10114–10123. 10.1007/s11356-016-6224-1. 26867689

[ref41] Kaneko H.; Shimada A.; Hirayama K. Short-term algal toxicity test based on phosphate uptake. Water Res. 2004, 38, 2173–2177. 10.1016/j.watres.2004.02.006. 15087199

[ref42] Silva N. F. P.; Gonçalves A. L.; Moreira F. C.; Silva T. F. C. V.; Martins F. G.; Alvim-Ferraz M. C. M.; Boaventura R. A. R.; Vilar V. J. P.; Pires J. C. M. Towards sustainable microalgal biomass production by phycoremediation of a synthetic wastewater: A kinetic study. Algal Res. 2015, 11, 350–358. 10.1016/j.algal.2015.07.014.

[ref43] Caporgno M. P.; Trobajo R.; Caiola N.; Ibañez C.; Fabregat A.; Bengoa C. Biogas production from sewage sludge and microalgae co-digestion under mesophilic and thermophilic conditions. Renew. Energy 2015, 75, 374–380. 10.1016/j.renene.2014.10.019.

[ref44] Mussgnug J. H.; Klassen V.; Schlüter A.; Kruse O. Microalgae as substrates for fermentative biogas production in a combined biorefinery concept. J. Biotechnol. 2010, 150, 51–56. 10.1016/j.jbiotec.2010.07.030. 20691224

[ref45] Thorin E.; Olsson J.; Schwede S.; Nehrenheim G. Co-digestion of sewage sludge and microalgae—Biogas production investigations. Appl. Energy 2018, 227, 64–72. 10.1016/j.apenergy.2017.08.085.

[ref46] Li L.; Kong X.; Yang F.; Li D.; Yuan Z.; Sun Y. Biogas production potential and kinetics of microwave and conventional thermal pretreatment of grass. Appl. Biochem. Biotechnol. 2012, 166, 1183–1191. 10.1007/s12010-011-9503-9. 22205322

[ref47] Wang M.; Lee E.; Dilbeck M. P.; Liebelt M.; Zhang Q.; Ergas S. J. Thermal pretreatment of microalgae for biomethane production: Experimental studies, kinetics and energy analysis. J. Chem. Technol. Biotechnol. 2017, 92, 399–407. 10.1002/jctb.5018.

[ref48] Syaichurrozi I.; Suhirman S.; Hidayat T. Effect of initial pH on anaerobic co-digestion of *Salvina molesta* and rice straw for biogas production and kinetics. Biocatal. Agric. Biotechnol. 2018, 16, 594–603. 10.1016/j.bcab.2018.10.007.

[ref49] Donoso-Bravo A.; Perez-Elvira S. I.; Fernández-Polanco F. Application of simplified models for anaerobic biodegradability tests. Evaluation of pre-treatment processes. Chem. Eng. J. 2010, 160, 607–614. 10.1016/j.cej.2010.03.082.

[ref50] Zhang Y.; Caldwell G. S.; Zealand A. M.; Sallis P. J. Anaerobic co-digestion of microalgae *Chlorella vulgaris* and potato processing waste: Effect of mixing ratio, waste type and substrate to inoculum ratio. Biochem. Eng. J. 2019, 143, 91–100. 10.1016/j.bej.2018.12.021.

